# Review of *Anaerobic Parasitic Protozoa: Genomics and Molecular Biology *by C. G. Clark, P. J. Johnson and R. D. Adam

**DOI:** 10.1186/1756-3305-3-45

**Published:** 2010-05-21

**Authors:** Kevin M Tyler

**Affiliations:** 1 Infection and Immunity Group, BioMedical Research Centre, School of Medicine, Health Policy and Practice, University of East Anglia, Norwich, Norfolk, UK

## Review

We live in an era of information technology, where sequencing is cheaper and easier than ever before and consequently the landscape of genomics and molecular biology is data rich and highly dynamic. Publication mediums are evolving rapidly in order to try to keep scientists everywhere up to date with the extensive datasets which are accruing. Into this melee, a beautifully produced and attractively packaged hardbound book of authoritative edited reviews seems at first glance anachronistic. Such reviews can be the subject of generic criticisms, that they serve only as a snapshot and therefore have a short shelf-life, which may be further shortened by the limited distribution of a specialist text. Further, since they are not in an electronic format the reviews cannot be hotlinked to the database resources from which they draw and are less accessible for translation by non-English speakers. Importantly, in terms of public accessibility and public engagement, this sort of publication format side-steps the open access requirement for publication by most major funders of tropical and neglected disease research and limits developing world access.

What mitigates against these concerns, in the case of this book, is its subject matter. Even amongst neglected diseases trichomoniasis, giardiasis and amoebiasis are significant in causing some of the highest morbidity, and indeed significant mortality, but receiving some of the least attention from the scientific community. For each of these fascinating and evolutionarily disparate organisms the genomes have been recently completed and thus a stage has been reached in their research history that can perhaps be best described as "the end of a beginning". Thus, this book at its best is a timely call to arms. The grouping of the three genome chapters at the beginning of the book is particularly helpful for ease of comparisons. Themes and peculiarities of evolutionary biology abound making it essential shelf material for those interested in the deep roots of the tree of life and the evolution of eukaryotic cell biology, cytoskeleton, mitochondria, genomic organization, gene expression, sex and speciation. The book is very well written and edited and so is easy to read even for non-specialists. By emphasising the progress made and the tools available for studying these organisms, as well as their interesting and often unique features, the book advocates for parasitologists, protozoologists and indeed all manner of microbiologists to look again at these important agents of disease as subjects of fascinating, worthwhile and tractable research.

## Competing interests

The author serves on the Advisory Board of the Open Access Journal "Parasites and Vectors."

